# On Glaucomatous Affections, and Their Treatment by Iridectomy

**Published:** 1865-02

**Authors:** W. Bowman


### *On Glaucomatous Affections and their Treatment by Iridectomy.* By W. BOWMAN, ESQ., F. R. S.

Before the British Medical Association, the author, after eloquently calling attention to the vast revolution which had been worked in the diagnosis and treatment of diseases of the eye since the invention of the opthalmoscope, by Helmholtz, passed on to the consideration of the treatment of glaucoma by iridectomy. He most warmly eulogized Von Graefe for having discovered a cure for a disease which but six or seven years ago was deemed incurable, and for having enriched the literature of the profession by his admirable papers on glaucoma, &c. Mr. Bowman then proceeded to give a masterly *expose* of the different symptoms of glaucoma in its different stages, viz:—the premonitory, acute, subacute, and chronic, and laid particular stress upon the increase of tension or hardness of the eye-ball—a symptom which he considers of paramount importance in all intraocular affections, and one which demands close and anxious watching. He also pointed out the fact noticed by Von Graefe, that a glaucomatous condition of the eye may supervene upon other affections of the eye, or after operation for cataract. The treatment required in such cases is an early iridectomy. He strongly urged the necessity of all medical men making themselves conversant with the various symptoms of glaucoma, in order that by a timely iridectomy eyes might be saved which were now irretrievably lost from the fact that the time had passed by when an iridectomy would have produced any improvement of sight. How often had we not to pass the melancholy verdict—"Too late! had you applied sooner your eye-sight might have been saved." Mr. Bowman vindicated the efficacy of irridectomy as a cure for glaucoma, having himself had many brilliant results of this operation. In conclusion, he explained the method of performing the different steps of the operation, the plan of incision, &c.

Dr. Richardson said he had been of late trying to work out the ynthesis of glaucoma in the same way as the synthesis of cataract, by the increase of the specific gravity of the blood. He should be glad to know if Mr. Bowman could tell, with absolute precision, what was the source of the fluid that produced the tension, and the nature of it; the source from which it was derived, and in what part of the eye-ball the tension existed; also, if he could explain physiologically the action of iridectomy. He had seen cases with such extraordinary results, that it was difficult to know how the operation really acted. He believed Mr. Bowman had somewhat under-estimated the blood conditions leading to glaucoma. It was necessary to consider that there was a gouty, or rheumatic, or super-fibrinized state of the blood, but a change simply in the specific gravity of the blood. By reducing the specific gravity of the blood, conditions were produced analogous to those occuring in glaucoma; so that, as cataract often depended upon an increase of specific gravity, the opposite effect might be produced by the opposite condition of the blood. He should be glad to know if Mr. Bowman had ever seen glaucoma in diabetes; for, therapeutically, the two diseases seemed incompatible.

Mr. Wells said he had seen the operation performed by its originator, Von Graefe, and could endorse all that Mr. Bowman had said respecting it. The reason why it had not been successful in some cases, were, that they had not been properly diagnozed; that the operation was not performed sufficiently early; or, that the mode of operation was not what it should have been—the incision not being made through the sclerotic.

Mr. Bowman said he came as a practitioner to speak to practitioners, and had therefore avoided going into anything like abstract or physiological reasoning. In one case he had seen the connection referred to by Dr. Richardson; but in that case there was a large amount of sugar, and the specific gravity was not higher than 1025. It was a case of chronic glaucoma—the sight of one eye was entirely gone and but little remained in the other. He performed the operation of iridectomy, and nothing could be more satisfactory than the recovery of the patient. With regard to the blood conditions, there might be varied morbid conditions of the blood not marked by albuminuria, or by anything gouty, rheumatic, or diabetic in the system. There was much more to be discovered on the subject by future observers, and there might be states of blood found to be intimately connected with glaucoma; but he thought the symptoms were rather referable to a local condition of the blood vessels and the nervous coats of the blood vessels in the eye. With regard to the *rationale* of the operation, he followed his friend Von Graefe. He at first thought that the result was produced by opening a freer communication between the back and front of the iris, thus allowing the fluids from behind to percolate more freely into the aqueous humour. He was not prepared to agree with Von Graefe's early supposition, that it might depend upon a removal of a portion of the iris, the iris being considered as the secreting agent of the aqueous humour, and that thus there might be a diminished secretion of the aqueous humour in the eye. He did not think it depended upon the quantity of iris removed; but upon the exactitude with which the marginal part of the iris was turned away from the ciliary bodies. With regard to the cause of the tension, he believed that in chronic cases it was an enlargement of the vitreous chamber of the eye, and in many cases a partial consolidation of the substance of the vitreous humour, not necessarily connected with any serum, or fibrous, or cloudy flocculi.

